# Pre-training, personalization, and self-calibration: all a neural network-based myoelectric decoder needs

**DOI:** 10.3389/fnbot.2025.1604453

**Published:** 2025-07-28

**Authors:** Chenfei Ma, Xinyu Jiang, Kianoush Nazarpour

**Affiliations:** School of Informatics, The University of Edinburgh, Edinburgh, United Kingdom

**Keywords:** adaptation, myoelectric control, neural network, deep learning, transfer learning

## Abstract

Myoelectric control systems translate electromyographic signals (EMG) from muscles into movement intentions, allowing control over various interfaces, such as prosthetics, wearable devices, and robotics. However, a major challenge lies in enhancing the system's ability to generalize, personalize, and adapt to the high variability of EMG signals. Artificial intelligence, particularly neural networks, has shown promising decoding performance when applied to large datasets. However, highly parameterized deep neural networks usually require extensive user-specific data with ground truth labels to learn individual unique EMG patterns. Meanwhile, the characteristics of the EMG signal can change significantly over time, even for the same user, leading to performance degradation during extended use. In this work, we propose an innovative three-stage neural network training scheme designed to progressively develop an adaptive workflow, improving and maintaining the network performance on 28 subjects over 2 days. Experiments demonstrate the importance and necessity of each stage in the proposed framework.

## 1 Introduction

Interaction with smart platforms, including wearable devices, is increasingly mediated by non-contact input. The AI Pin exemplifies a technology that has moved beyond traditional hardware and touch-screen interaction. Low-cost biomedical sensing, such as electromyography (EMG) has shown great promise in enhancing these non-contact input methods. Generation of EMG signals involves complex processes in which brain signals trigger electrical impulses in muscles, leading to muscle contraction, and generating an electrical field detected by EMG electrodes from the surface of the skin. Coupled with inertial sensing, EMG has been shown to be capable of reliably detecting motion intentions (labs at Reality Labs et al., 2024).

Modern myoelectric control has traditionally relied on pattern recognition techniques to interpret muscle signals (Asghar et al., 2022) for prosthetic and wearable device operations. These methods involve extracting features from EMG signals and classifying them using linear or non-linear models (Asghar et al., 2022). Although exciting, traditional pattern recognition approaches often struggle with the variability of the EMG signals and therefore fall short of recognizing complex hand gestures reliably.

### 1.1 Neural network for myoelectric control—a précis

Recently, there has been a significant shift toward the use of modern machine learning techniques, particularly neural networks, for myoelectric control (Fleming et al., 2021; Khushaba and Nazarpour, 2021; Hu et al., 2023). Neural networks, with their ability to model non-linear input-output relationships and learn from large datasets, offer a more personalized and adaptable solution. This transition has the potential to enhance the performance and reliability of myoelectric control, paving the way for more intuitive human-machine interactions and better user experiences.

These neural network-based innovations build upon decades of research in the myoelectric control of bionic limbs. For example, Kelly et al. (1990) used multilayer perceptron neural networks in myoelectric control. They laid the foundation for research on machine learning and deep learning-driven techniques in myoelectric control. Au and Kirsch (2000) applied a time-delayed artificial neural network to an EMG-based shoulder movement estimation task, which showed the importance of including information from previous time steps. Bu et al. (2003) and Song and Tong (2005) embedded recurrent structures in the neural network for the estimation of movement and the estimation of elbow torque, respectively. Modern 2D convolutional neural networks (CNN) (Park and Lee, 2016; Atzori et al., 2016), temporal convolutional networks (TCNs) (Betthauser et al., 2019), long-short-term memory (LSTM) neural networks (Teban et al., 2018), together with their variants (Rahimian et al., 2021; Godoy et al., 2022; Liu et al., 2023; Ma et al., 2020, 2021) and combinations (Xia et al., 2018; Jabbari et al., 2021, 2020), have shown promising performance in EMG decoding. In addition, transfer learning protocols and adversarial neural networks in the adaptive domain, for example, were utilized to build generalizable and cross-user models (Campbell et al., 2021), as well as user-independent models (Côté-Allard et al., 2020). General domain adaptation algorithms have also been well examined in previous studies to adapt EMG variabilities caused by various factors (Lin et al., 2023; Zhang et al., 2023, 2022; Shi et al., 2022; Tam et al., 2021; Ameri et al., 2019).

Despite their success, most of the previous methods have remained academic curiosities and are yet to be translated into extended-reality or myoelectric control applications. For instance, most of the above models were trained with data from the same user, increasing the risks of overfitting (Wang and Buchanan, 2002), limiting the generalizability across new users, and showing less robustness to non-stationarities. Such a user-specific model also requires a large amount of training data from the target user, increasing the data collection burden on each user. Furthermore, simply combining personalized data from the target user and generalized data from others to train a large model also raises privacy concerns about data sharing (Zhang et al., 2017). In addition to variability between users, the characteristics of the EMG of the same user can change substantially over time due to factors such as behavior change (Ludwig, 1982) or fatigue (Dimitrova and Dimitrov, 2003). Consequently, an ideal myoelectric model should be generalizable, adaptive, privacy safe, and self-improving.

In this paper, we introduce a paradigm for neural network-based myoelectric control which

1. is *pre-trained* by data from many participants, taking a step toward future between-user model generalization.
2. enables the *personalization* of the pre-trained model to a new user with a small amount of data that only includes one trial per movement.
3. *self-calibrates* autonomously to adapt to changing myoelectric behavior of the user.

We demonstrated progressive performance improvements by including each of the key modules above. We hope that our work can provide a useful framework for neural network training in future practical real-time myoelectric control applications.

## 2 Methods

**Figure 1:**
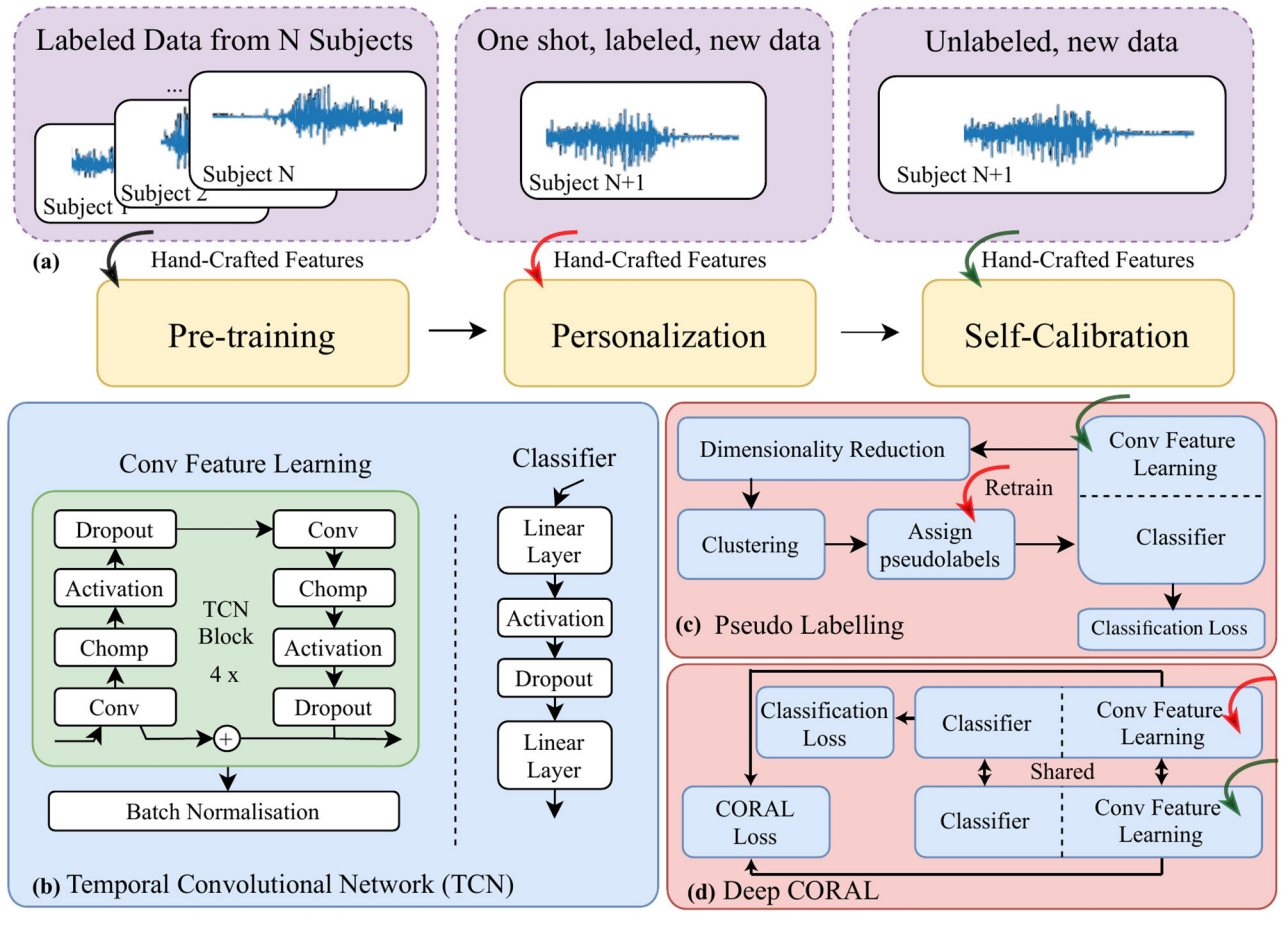
A block diagram for **(a)** the data pipeline, “one shot” stands for only one trial of movement being included **(b)** the architecture of a temporal convolutional network; **(c)** self-calibration via pseudo-labeling; and **(d)** self-calibration via a modified deep CORAL to further align data distribution in the latent space projected by the feature learning module.

Figure 1 shows the block framework of the proposed method. Eight hand-crafted features, namely waveform length (WL), log variance (LV), zero crossing (ZC), slope sign changes (SSC), skewness (SKW), mean frequency (MNF), peak frequency (PKF), and variance of central frequency (VCF), a similar choice to our previous work (Jiang et al., 2024a, 2025), were extracted from training EMG data with a window size of 150 ms and a stride of 5 ms. The features were segmented by another sliding window with a window size of 250 ms and stride of 50 ms for use in pre-training, personalization, and self-calibration steps. In the following, we detail each compartment.

### 2.1 Pre-training

Pre-training refers to the process of initializing a model on a large dataset to learn general features before fine-tuning it on a more specific task or a smaller dataset (Hendrycks et al., 2019). Using pre-trained networks addresses a key challenge in myoelectric control, which is minimizing the risk that a small sample size biases the training of a network. Furthermore, pre-training enhances the possibility of enhancing the generalization ability.

The structure of the temporal convolutional neural network (TCN) was designed to analyze temporal information, for example, for video processing (Lea et al., 2017). The key advantage of TCNs over conventional CNNs is the combination of dilated and causal convolutions, which expands the neural network's receptive field and focuses on relevant data for the current time step. This feature makes TCNs particularly useful for myoelectric signal processing and movement classification, as shown in our previous work (Ma and Nazarpour, 2024). We therefore chose TCN as the backbone of our approach. In particular, we adopted a 4-block TCN, as illustrated in Figure 1b, to improve model portability and transparency. The network has two high-level blocks, namely feature learning and the classifier. We have separated these two in Figure 1a as we deal with them separately at a later stage in the framework.

The classification accuracy of this pre-trained model serves as a benchmark in this study. We build upon it with additional neural network blocks, namely, personalization and self-calibration.

### 2.2 Personalization

After pre-training the model with the collected base dataset, the model could learn general knowledge. However, individual differences in EMG data distribution usually lead to the degradation of model performance when applying a pre-trained model to new users. The personalization stage in the framework could adjust the weights of the neural networks without disrupting the pre-built structure. This stage aligns the model with the shifted data distribution of the new user. Diverse methods could be utilized to align the model, e.g., fine-tuning (Bengio, 2012), data selection (Afridi et al., 2018; Ruder and Plank, 2017), domain adaptation (Kouw and Loog, 2018), and miscellaneous transfer learning methods (Tan et al., 2018).

In our work, we chose the fine-tuning method (Bengio, 2012), as a proof of principle, which took the parameters from the pre-trained model as a starting point and then further updated these parameters by backpropagation (Hecht-Nielsen, 1992). Importantly, we employed labeled data from only one trial (1 s duration) per class to fine-tune the pre-trained model, demonstrating that the personalization process can be achieved in a highly data-efficient way. Specifically, the weights for each TCN layer were unfrozen and fine-tuned using the Adam optimizer (Kingma and Ba, 2014) with a gradient descent on the cross-entropy loss (Shannon, 1948).

### 2.3 Self-calibration

Personalization partially adapts the model to the new user. However, the user's evolving myoelectric behavior over time causes performance variability because the model remains static while the behavior changes. In addition, the skin condition, electrode repositioning, etc. also extend the challenge. This highlights the need for a system that continuously updates, keeping the model adaptive. Figures 1c, d illustrate the two methods that we adopted for self-calibration.

#### 2.3.1 Self-calibration via pseudo-labeling

We first adopted a naive approach (Jiang et al., 2024b), assuming that the characteristics of the EMG signal distribution change slowly. Therefore, we can retrain the neural network by assigning pseudo-labels to the incoming testing EMG data. Specifically, we utilized (1) t-distributed Stochastic Neighbor Embedding (t-SNE) (Hinton and Roweis, 2002), a dimensionality reduction method enabling manifold learning, and (2) K-means (Hartigan and Wong, 1979), a clustering algorithm, to jointly create pseudo-labels. The pseudo-labels were then used to update and retrain the neural network in the background. The block diagram illustrating this process is shown in Figure 1c.

Note that during pseudo-labeling, we first froze the feature learning module of the neural network and then input the EMG features of the unlabeled data into the feature learning module. The output variables (features in the latent space) were then fed into t-SNE and then K-means algorithms to get the pseudo-labels. The K-means algorithm was initialized as the prediction outcomes of the personalized model. Following clustering, one trial labeled data was used to align the pseudo-labels with the correct labels. Then the pseudo-labeled data was used to retrain the classifier. This process occurs periodically when a certain number of samples per label is collected during the operation, thus maintaining the adaptability of the model.

#### 2.3.2 Domain adaptation via deep CORAL

Another approach to enable self-calibration is to match the distribution of new unlabeled sEMG data (target domain) with the previously collected labeled data (source domain). We therefore used deep correlation alignment (CORAL), an unsupervised domain adaptation method (Sun and Saenko, 2016). This method perfectly suits cases where the target domain and source domain share similar features and label space, but the distributions are different. CORAL (Sun et al., 2016), similar to maximum mean discrepancy (MMD) (Gretton et al., 2012), is a measure of distribution divergence between observed samples.

Suppose that the training samples in the source domain (one trial labeled data) are denoted as $D_{s}=\left\{x_{i}\right\},x\in {\mathbb{R}}^{d},i\in \left\{1,2,...,n_{S}\right\}$, corresponding to labels *L*_*s*_ = {*y*_*i*_}, *i*∈{1, 2, ..., *n*_*S*_}, with the sample number of *n*_*S*_, and the unlabeled data samples (from the self-calibration stage) in the target domain are $D_{t}=\left\{z_{i}\right\},z\in {\mathbb{R}}^{d},i\in \left\{1,2,...,n_{T}\right\}$, with the sample number of *n*_*T*_. The feature covariance matrices of the source domain *C*_*S*_ and the target domain *C*_*T*_ could be calculated as:


(1) $$\begin{array}{l} C_{S}=\frac{1}{n_{S}-1}\left(D_{S}^{⊤}D_{S}-\frac{1}{n_{S}}{\left(1^{⊤}D_{S}\right)}^{⊤}\left(1^{⊤}D_{S}\right)\right) \end{array}$$

(2) $$\begin{array}{l} C_{T}=\frac{1}{n_{T}-1}\left(D_{T}^{⊤}D_{T}-\frac{1}{n_{T}}{\left(1^{⊤}D_{T}\right)}^{⊤}\left(1^{⊤}D_{T}\right)\right) \end{array}$$

where **1** is a column vector filled with elements of 1. After the covariance computation in both the source and target domains, the CORAL loss, based on the distance between the covariances of both domains, could be presented as follows:


(3) $$\begin{array}{l} \ell_{CORAL}=\frac{1}{4d^{2}}||C_{S}-C_{T}|{|}_{F}^{2} \end{array}$$

where $||\cdot |{|}_{F}^{2}$ stands for the Frobenius norm. In the context of self-calibration, the source domain data and target domain data are from the same (new) user.

Conventional deep CORAL minimizing the CORAL loss alone is very likely to degenerate the feature learning outcome because simple features from different classes are very likely to overlap together to reduce the total CORAL loss. Meanwhile, only minimizing the classification loss in the source domain can lead to overfitting, increasing the domain shift between the source and target domains. Since the variables in the latent space given by the feature learning module should be discriminative enough in both the source and target domains, minimizing the CORAL loss on the output of the feature learning module is a promising solution. We therefore minimized a hybrid training loss as follows:


(4) $$\begin{array}{l} \ell =\ell_{classification}+\lambda \ell_{CORAL} \end{array}$$

where λ is a parameter that trades off different loss functions.

### 2.4 Ethics

All experimental procedures were conducted in accordance with the Declaration of Helsinki and were approved by the local Ethics Committee of the School of Informatics at the University of Edinburgh (2019/89177). All participants read an information sheet and gave their consent prior to the experiments. A total of 28 participants, aged between 21 and 43 years, including 13 males and 15 females, were recruited for this study.

### 2.5 Data collection experiment

**Figure 2:**
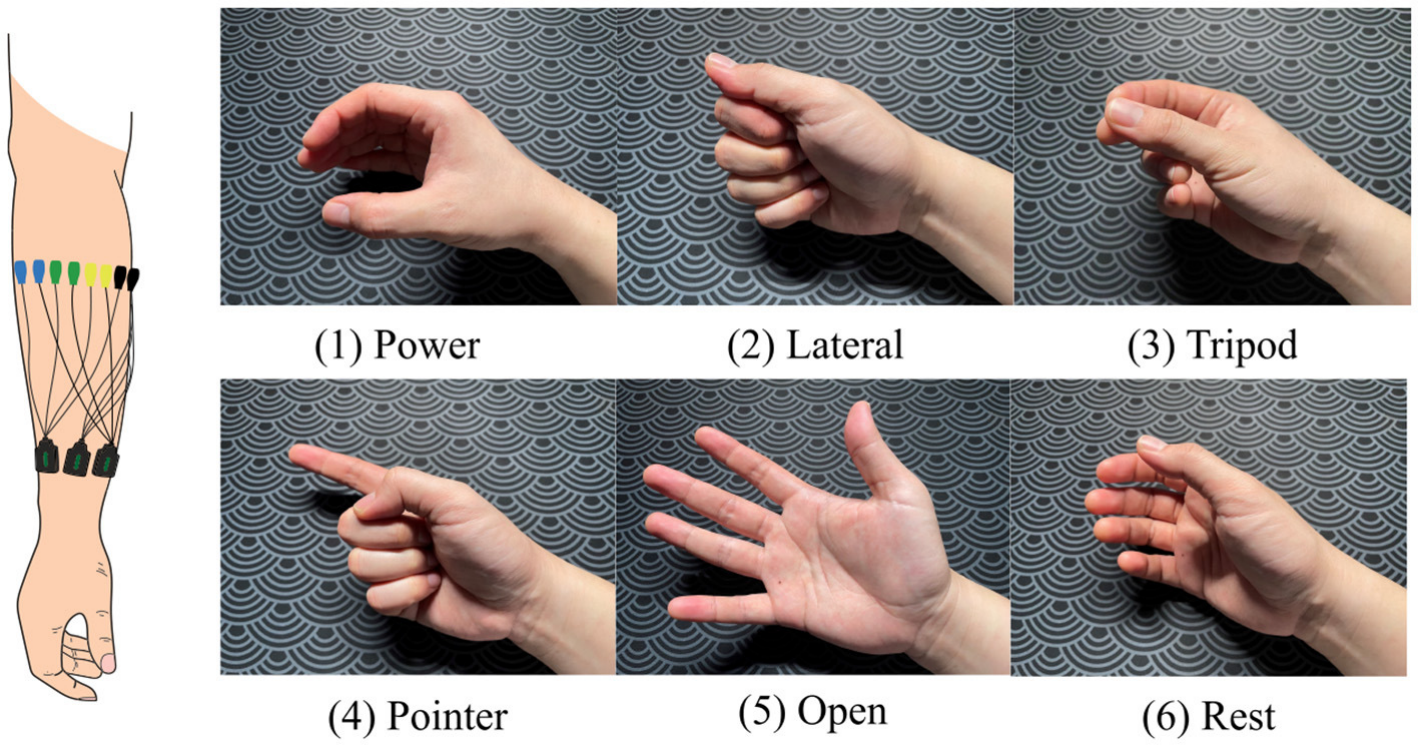
The electrode placement and all the movements comprised in the research.

Fifteen EMG sensors (Delsys Trigno, USA) were placed around the forearm of the dominant arm, 2 cm below the elbow, starting from the extensor carpi ulnaris muscle on each participant for data collection (Figure 2). After preparation, each participant was instructed to perform one trial per gesture, following the on-screen instructions during the data collection phase. These data (one trial per hand gesture) were used for model personalization. The gestures included power, lateral, tripod, pointer, open, and rest (Figure 2).

For each of the 28 participants, data from 2 days was collected. On the first day, a calibration session was first conducted, with one trial per hand gesture. Each trial is of 2 s duration, and participants shape their hand in the first second and holding the same hand gesture in the last second. A 2-s inter-trial interval was provided. In all data collection of the 28 participants, we followed the same trial duration and inter-trial interval. Data collected in the calibration session was used to personalize the model in one shot. After the calibration session on the first day, five test blocks were performed, with 30 trials per block. Therefore, we collected 150 trials for all 6 hand gestures, that is, 25 trials per gesture. Participants could take flexible self-paced breaks between test blocks, typically 5 min. On the second day, participants directly started five more testing blocks without any calibration session. Each test block lasted about 2 min and the total duration of the experiment on each day was 40 min, including intervals. Labels are balanced for each day. By exploring the performance variation along all test blocks on the same day and on 2 days, we could compare the robustness of different models during long-term use.

Data were sampled at 2,000 Hz and filtered using a 4th-order bandpass Butterworth filter with a frequency range of 10 to 500 Hz. After collecting data for personalization, participants were guided through 10 blocks of tests without feedback. In order to verify its robustness on temporal variance, trials were reordered into five randomly ordered trials for each gesture, totaling 30 trials per block. During each trial, all participants were instructed to perform one hand gesture as displayed on the screen for 2 s while the data and labels were recorded. To account for individual differences in reaction times, only the data from the latter 1-s interval were used for analysis. The decision to withhold feedback from participants was made to prevent bias in their behavior.

### 2.6 Validation methods

We tested four different models: the pre-trained model, the personalized model, the self-calibrated model via pseudo-labeling, and the self-calibrated model via deep CORAL. It is important to note that each of the latter models includes all the key modules from the previous ones. For example, the personalized model indicates that the model has been pre-trained prior to personalization. Since self-calibration involves block-wise training (the model would be trained/calibrated over each block), for a fair comparison, performance testing was conducted only on the last two trials of each class in each block; the first three trials of each class were used for self-calibration, which requires block-wise training.

In the pre-training step, leave-one-person-out cross-validation was applied. This meant that we repeated the analysis 28 times, using data from 27 participants to pre-train the model, while the data from the held-out participant was used for personalization and subsequent testing of the model. The accuracy of the prediction for the movement labels, compared to the recorded ground truth in each sliding window, was used to evaluate model performance.

Training and testing on a workstation with an AMD Ryzen Threadripper PRO 3975WX 32-Cores CPU and two NVIDIA GeForce RTX 3090 GPUs in parallel, in a Python 3.8.10 environment.

### 2.7 Data augmentation

Data augmentation is one of the most commonly adopted data preprocessing techniques in deep learning (Mumuni and Mumuni, 2022). However, unlike images, myoelectric signals do not have explicit or structured patterns, which means that they cannot be scaled or rotated. Therefore, we created a virtual channel between each pair of adjacent physical channels by averaging the signal values. This step increased the number of channels from 15 to 30.

## 3 Results

**Figure 3:**
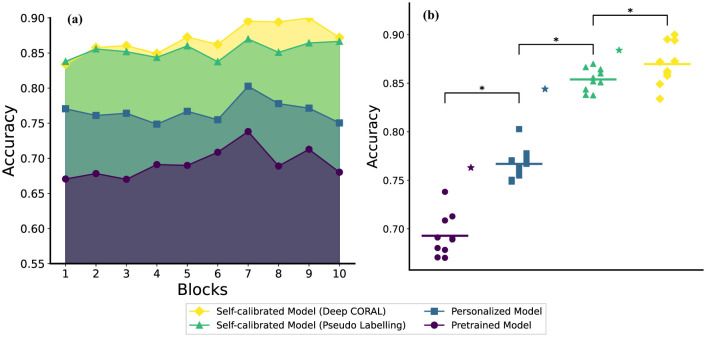
The comparison figure on **(a)** The test accuracy (average across subjects) results on each block **(b)** The statistical results of each method (average across subjects) on stages and the stars with the same color represent the corresponding stage results for random forest (RF) method which are calculated using the method presented by Jiang et al. (2024a,b, 2025) (the two sub-figures share the same legend).

As illustrated in Figure 3a, the personalized model outperforms the pre-trained model across all 10 blocks. Additionally, the self-calibrated models—achieved through both the pseudo-labeling method and the deep CORAL method—outperform the personalized model. Specifically, the deep CORAL method contributes to superior performance in most test blocks compared to the pseudo-labeling method.

In terms of performance, the pre-trained model stays in a relatively low standard range, personalized models, which performed better, experienced a decrease at the beginning of the testing process, while the self-calibration models showed better results and kept an increasing trend. Furthermore, users naturally varied their behavior when performing hand gestures as the experiment progressed, leading to fluctuations in model performance. Notably, after block 7, the pre-trained and personalized model exhibited significantly more unstable accuracy than other models, as shown in Figure 3a.

To validate the hypothesis that the module at each stage of our training process is necessary, first we carried out the Friedman test, which proved there was an overall effect of decoder choice (χ^2^ = 75.4, *p* < 10^−16^). We then performed statistical analyzes (Wilcoxon Signed Rank test) between each pair of models. Because three comparisons were made, the Bonferroni correction was performed. The results after correction are presented in Figure 3b. The personalized model (accuracy: 0.767 ± 0.016) significantly (*p* = 0.006) outperforms the pre-trained model (accuracy: 0.693 ± 0.021). The pseudo-labeling-based self-calibration model (accuracy: 0.854 ± 0.012) significantly (*p* = 0.006) outperforms the personalized model. Similarly, the deep CORAL-based self-calibration model (accuracy: 0.870 ± 0.022) significantly (*p* = 0.018) outperforms the pseudo-labeling-based self-calibration model. We also included the random forest method in all 3 stages, the results for the pre-training, personalization, and self-calibration stages are 0.763, 0.844, and 0.884, respectively (Jiang et al., 2024a,b, 2025). The progressively improved accuracy demonstrates the necessity of each module in the proposed neural network training protocol.

**Figure 4:**
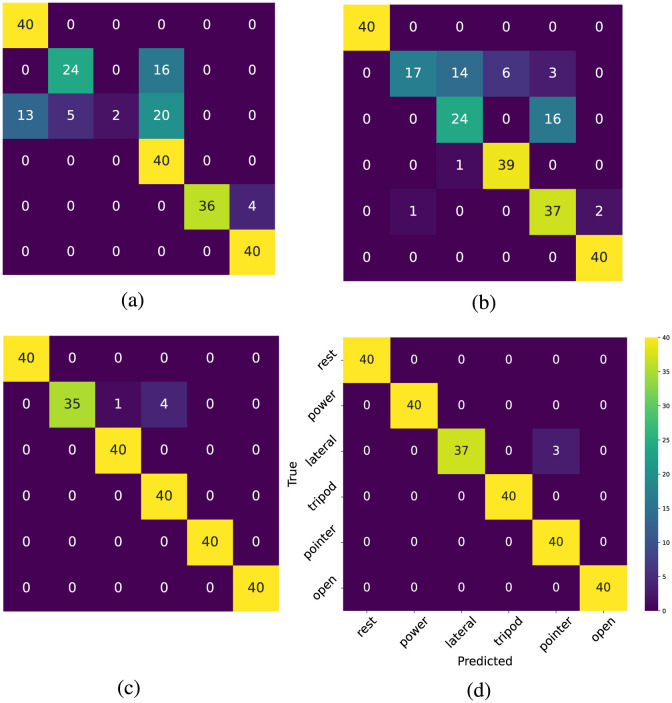
**(a)** Confusion matrix of the pre-trained model **(b)** Confusion Matrix of the personalized model **(c)** Confusion matrix of the self-calibrated model (pseudo-labeling) **(d)** Confusion matrix self-calibrated Model (deep CORAL).

To examine the performance of the model in recognizing each hand gesture, the confusion matrices for one subject in the last test block are presented in Figure 4. The number 40 is the number of classifications during the final two trials in the last test block. As shown in Figure 4a, the pre-trained model struggles to distinguish the lateral gesture. In Figure 4b, the personalized model has adapted to the user's pattern and can generally predict the movements. However, it still exhibits a low recall rate for power and lateral hand gestures. Figures 4c, d indicate that the self-calibration methods further enhance the model's performance across most classes.

**Figure 5:**
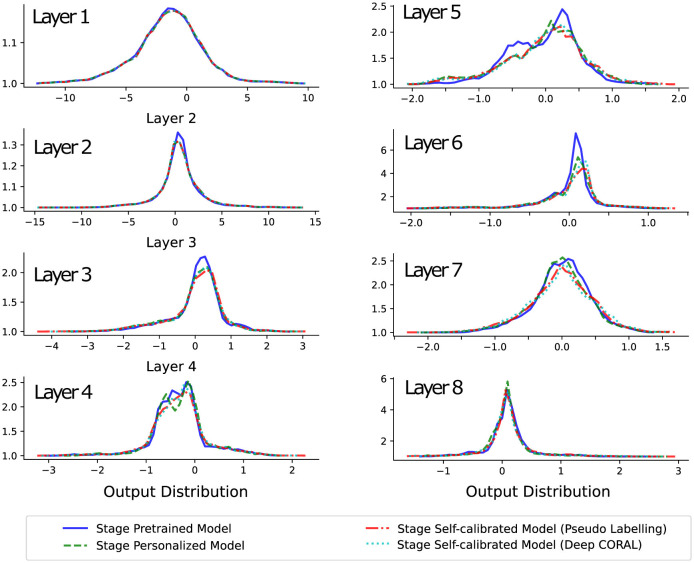
This plot demonstrates the distribution changes of output variables on the same model layers, over different stages. The values from the superficial layer to the deep layer show that the model extracts and abstracts the lower-dimensional high variant inputs into higher-dimensional low variant semantic information. Besides, the changes across stages present that the feature extraction (superficial layers) and feature space organization (deep layers) are somewhat constant, but the weights in the middle layers show considerable changes.

The distributions of the outputs from each convolutional layer for one subject in the final experiment test block are shown in Figure 5. Since the model has learned general feature representations during the pre-training stage, the behavior of the first layer's feature representation did not change significantly when the personalization and self-calibration modules were introduced.

From the second to the middle layers of the model, noticeable differences in output distribution can be observed across different models, with these differences being especially pronounced in the middle layers, such as layers 4 and 5. However, from the middle to the deepest layers, the output distributions of different models begin to overlap again. This suggests that the variation in performance between models is primarily due to differences in behavior in the middle layers.

## 4 Discussion

In this study, we proposed a three-stage neural network training protocol for myoelectric control models, consisting of pre-training, personalization, and self-calibration. Neural networks are increasingly applied in myoelectric control applications, but the standard one-stage training approach has significant limitations, particularly in terms of extensive data collection requirements and the inability to adapt during long-term use. Furthermore, privacy concerns surrounding personal biomedical data, coupled with the complexity of EMG signals and individual variability, make it challenging to directly leverage data from other users or even historical data from the same user. To address these issues, we introduce a multistage training scheme designed to mitigate these challenges and enhance model robustness.

### 4.1 Pre-training: reducing data collection burden

The pre-training stage serves as the foundation by leveraging previously collected datasets from a large number of users. This allows the model to learn general patterns in the EMG data, significantly reducing the burden of data collection for each new target user. In this stage, a common model is trained to extract generalizable features across users, encoding this knowledge into the network weights. However, despite these benefits, pre-training alone is insufficient for achieving robust performance in new users. As shown in Figure 3a, the pre-trained model, without any further calibration, performed the worst from the beginning of the testing phase and showed increasingly unstable performance from block 7. This is due to inherent factors such as variations in muscle conditions, electrode placement, and behavioral differences for the same movement, all of which degrade model performance over time.

### 4.2 Personalization: learning user-specific characteristics

To address this, we introduced the personalization stage, allowing the pre-trained model to adapt to the specific characteristics of a new user. In this stage, data from just one trial (1-s signal duration) per hand gesture are collected to fine-tune the model. This approach differs from traditional methods, where models are trained from scratch either with large datasets from other users or solely with data from the new user. By separating pre-training and personalization, our method establishes a foundational understanding of general EMG signals, which is then personalized using a minimal amount of new user data. Importantly, the pre-trained model's knowledge (on feature extraction, superficial layers of the network) is retained in its weights and parameters, allowing the personalization stage to adjust the model without needing access to the original pre-training data. This is crucial to address privacy concerns related to biomedical data. As demonstrated in our results, the personalized model significantly outperformed the non-personalized model and also exhibited greater stability than the pretrained model with better performance, highlighting the value of this stage in long-term applications.

### 4.3 Self-calibration: adapting to changes over time

However, the human motor system is inherently complex, leading to unpredictable muscle states, such as fatigue and behavioral variability over time. Individual differences in memory retention and movement execution can also cause significant changes in EMG patterns during long-term use, further reducing the robustness of the model. Frequent recalibration is often needed to maintain accuracy. To address this, we propose a self-calibration stage that uses unlabeled data collected during the model's inference process. This unlabeled data enables unsupervised self-calibration, allowing the model to adapt to the latest EMG data distributions and even learn new information to enhance its performance. Our results show that both self-calibration methods significantly improved performance compared to the personalized model without self-calibration (Figure 3a).

While self-calibration has received relatively limited attention in previous studies, it is essential for real-world applications. In our work, we implemented and compared two methods: deep CORAL and pseudo-labeling. Deep CORAL incorporates additional guidance by leveraging classification performance on a small number of labeled data points collected during the one-trial personalization stage. In contrast, pseudo-labeling relies on both the model from the previous update for prior knowledge and the one-trial data. The relatively better performance of deep CORAL demonstrates that transfer learning and domain adaptation, when applied in the self-calibration stage, can significantly improve model performance by utilizing both model parameters and previous data.

We have previously developed a flexible mechanism for self-calibrating random forests (Jiang et al., 2024a,b, 2025), which demonstrated superior generalization performance compared to the neural network approach evaluated in this work. As shown in Figure 3b, the random forest consistently outperforms the neural network at each stage. This is largely due to its robustness when working with limited datasets, as neural networks, by design, have a higher parameter count and greater capacity, making them prone to overfitting in low-data regimes. However, there are important trade-offs between the two approaches. Random forests offer simplicity, are more straightforward to implement in hardware, and perform reliably with smaller datasets. In contrast, neural networks provide greater flexibility and scalability when handling larger datasets, where their capacity to learn hierarchical representations becomes advantageous.

### 4.4 Limitations and future directions

Although our methods represent innovative approaches to improve myoelectric control models, there is still room for improvement. First, the pre-training dataset we used was relatively small. Previous research has shown that increasing the size and diversity of the pre-training dataset can dramatically enhance model performance, and we expect similar improvements with a larger dataset in future work.

Second, the personalization stage involved collecting only one trial per hand gesture at the beginning of the experiment. This limited amount of data may introduce bias or fail to capture variations in EMG signals over time. A more robust personalization process, potentially using more labeled data from the new user, could further improve the model's adaptability and accuracy in real-world settings.

Finally, during the self-calibration stage, we used unlabeled data from only three trials per movement in each test block. Although this was sufficient for demonstrating proof of concept, a larger data buffer in real-world applications could capture more information about the current EMG data distribution, leading to more effective model calibration.

## 5 Conclusion

Our study highlights the importance of a multi-stage training protocol for neural network-based myoelectric control models. The combination of pre-training, personalization, and self-calibration addresses key challenges related to data scarcity, privacy concerns, and long-term usability. While there is potential for improvement, particularly in expanding pre-training datasets and enhancing personalization and self-calibration methods, the results presented here provide a strong foundation for future work. These methods, when further refined, have the potential to significantly enhance the adaptability, robustness, and practicality of neural network-based myoelectric decoders in real-world applications.

## Data Availability

The raw data supporting the conclusions of this article will be made available by the authors, without undue reservation.
