# Correction: Pre-training, personalization, and self-calibration: all a neural network-based myoelectric decoder needs

**DOI:** 10.3389/fnbot.2025.1675642

**Published:** 2025-09-19

**Authors:** Chenfei Ma, Xinyu Jiang, Kianoush Nazarpour

**Affiliations:** School of Informatics, The University of Edinburgh, Edinburgh, United Kingdom

**Keywords:** adaptation, myoelectric control, neural network, deep learning, transfer learning

In the published article, in Figure 1, the EMG traces in Figure 1a were erroneously excluded, and the arrow in Figure 1d is deformed. The corrected Figure 1 appears below.

**Figure 1:**
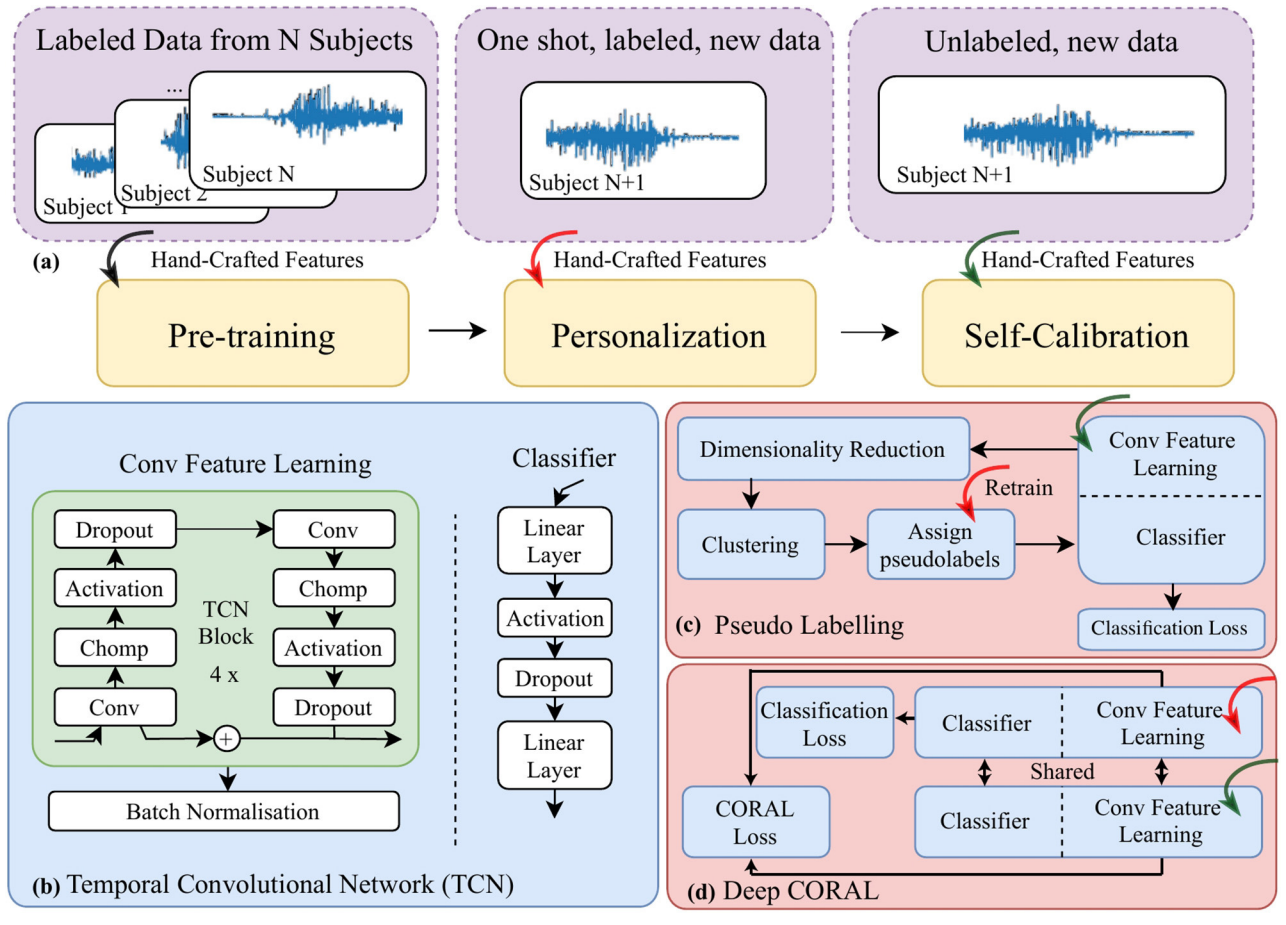
A block diagram for **(a)** the data pipeline, “one shot” stands for only one trial of movement being included **(b)** the architecture of a temporal convolutional network; **(c)** self-calibration via pseudo-labeling; and **(d)** self-calibration via a modified deep CORAL to further align data distribution in the latent space projected by the feature learning module.

In the published article, in the abstract, an incorrect word (“However”) is used in the sentence “However, the characteristics of the EMG signal can change significantly over time, even for the same user, leading to performance degradation during extended use.” The corrected sentence appears below:

“Meanwhile, the characteristics of the EMG signal can change significantly over time, even for the same user, leading to performance degradation during extended use.”

In the published article, in Section 2.5 **Data Collection**, paragraph 2, two sentences were not separated, and an incorrect word was used. The corrected paragraph appears below:

“For each of the 28 participants, data from 2 days was collected. On the first day, a calibration session was first conducted, with one trial per hand gesture. Each trial is of 2 s duration, and participants shape their hand in the first second and holding the same hand gesture in the last second. A 2-s inter-trial interval was provided. In all data collection of the 28 participants, we followed the same trial duration and inter-trial interval. Data collected in the calibration session was used to personalize the model in one shot. After the calibration session on the first day, five test blocks were performed, with 30 trials per block. Therefore, we collected 150 trials for all 6 hand gestures, that is, 25 trials per gesture. Participants could take flexible self-paced breaks between test blocks, typically 5 min. On the second day, participants directly started five more testing blocks without any calibration session. Each test block lasted about 2 min and the total duration of the experiment on each day was 40 min, including intervals. Labels are balanced for each day. By exploring the performance variation along all test blocks on the same day and on 2 days, we could compare the robustness of different models during long-term use.”

The original version of this article has been updated.

